# Computer vision applications for the detection or analysis of *tuberculosis* using digitised human lung tissue images - a systematic review

**DOI:** 10.1186/s12880-024-01443-w

**Published:** 2024-11-05

**Authors:** Kapongo D. Lumamba, Gordon Wells, Delon Naicker, Threnesan Naidoo, Adrie J. C. Steyn, Mandlenkosi Gwetu

**Affiliations:** 1 School of Mathematics, Statistics and Computer Science, University of Kwazulu Natal (UKZN), King Edward Avenue, Scottsville, Pietermaritzburg, 3209 KwaZulu Natal Republic of South Africa; 2 Africa Health Research Institute, UKZN, 719 Umbilo Road, Durban, 10587 KwaZulu Natal Republic of South Africa; 3 Department of Microbiology, University of Alabama at Birmingham, 1720 2nd Ave South, Birmingham, 35294 AL USA; 4 Department of Forensic and Legal Medicine, Walter Sisulu University, Nelson Mandela Dr, Umtata Part 1, Mthatha, 5099 Eastern Cape Republic of South Africa; 5 Department of Industrial Engineering, Stellenbosch University, Faculty of Engineering, Banghoek Rd, Stellenbosch, Western Cape 7600 Republic of South Africa

**Keywords:** Human lung tissue, *Tuberculosis*, Image analysis, Deep learning

## Abstract

**Objective:**

To conduct a systematic review of the computer vision applications that detect, diagnose, or analyse *tuberculosis* (TB) pathology or *bacilli* using digitised human lung tissue images either through automatic or semi-automatic methods. We categorised the computer vision platform into four technologies: image processing, object/pattern recognition, computer graphics, and deep learning. In this paper, the focus is on image processing and deep learning (DL) applications for either 2D or 3D digitised human lung tissue images. This review is useful for establishing a common practice in TB analysis using human lung tissue as well as identifying opportunities for further research in this space. The review brings attention to the state-of-art techniques for detecting TB, with emphasis on the challenges and limitations of the current techniques. The ultimate goal is to promote the development of more efficient and accurate algorithms for the detection or analysis of TB, and raise awareness about the importance of early detection.

**Design:**

We searched five databases and Google Scholar for articles published between January 2017 and December 2022 that focus on *Mycobacterium tuberculosis* detection, or *tuberculosis* pathology using digitised human lung tissue images. Details regarding design, image processing and computer-aided techniques, deep learning models, and datasets were collected and summarised. Discussions, analysis, and comparisons of state-of-the-art methods are provided to help guide future research. Further, a brief update on the relevant techniques and their performance is provided.

**Results:**

Several studies have been conducted to develop automated and AI-assisted methods for diagnosing *Mtb* and TB pathology from digitised human lung tissue images. Some studies presented a completely automated method of diagnosis, while other studies developed AI-assisted diagnostic methods. Low-level focus areas included the development of a novel $\upmu$CT scanner for soft tissue image contract, and use of multiresolution computed tomography to analyse the 3D structure of the human lung. High-level focus areas included the investigation the effects of aging on the number and size of small airways in the lungs using CT and whole lung high-resolution $\upmu$CT, and the 3D microanatomy characterisation of human *tuberculosis* lung using $\upmu$CT in conjunction with histology and immunohistochemistry. Additionally, a novel method for acquiring high-resolution 3D images of human lung structure and topology is also presented.

**Conclusion:**

The literature indicates that post 1950s, TB was predominantly studied using animal models even though no animal model reflects the full spectrum of human pulmonary TB disease and does not reproducibly transmit *Mtb* infection to other animals (Hunter, 2011). This explains why there are very few studies that used human lung tissue for detection or analysis of *Mtb*. Nonetheless, we found 10 studies that used human tissues (predominately lung) of which five studies proposed machine learning (ML) models for the detection of *bacilli* and the other five used CT on human lung tissue scanned ex-vivo.

## Introduction

*Tuberculosis* (TB) is an infectious disease that has been known for thousands of years and is caused by *Mycobacterium tuberculosis* (*Mtb*), which primarily affects human lungs and was first identified by Robert Koch in 1882 [3, 7]. The bacteria mainly attack the alveoli and surrounding tissue, causing inflammation and damage to the lung tissue. The susceptibility of humans to TB can be traced back 9,000 years ago as per the findings from the archaeological remains in the ancient city of Atlit Yam, off the coast of Israel [3]. For more than a century, histopathological analysis depended almost entirely on two-dimensional (2D) plane for investigation and diagnosis of TB [13]. “One would think that the pathology of *tuberculosis* would have been accurately described long ago and that science in the 21st century would have advanced far beyond morphologic descriptions” [11], but such is not the case. Despite considerable effort, we understand little about what distinguishes individuals who progress to active TB from those who remain latent for decades [12]. According to the World Health Organization (WHO), *Mtb* is responsible for over 1.30 million deaths globally [31].

**Fig. 1:**
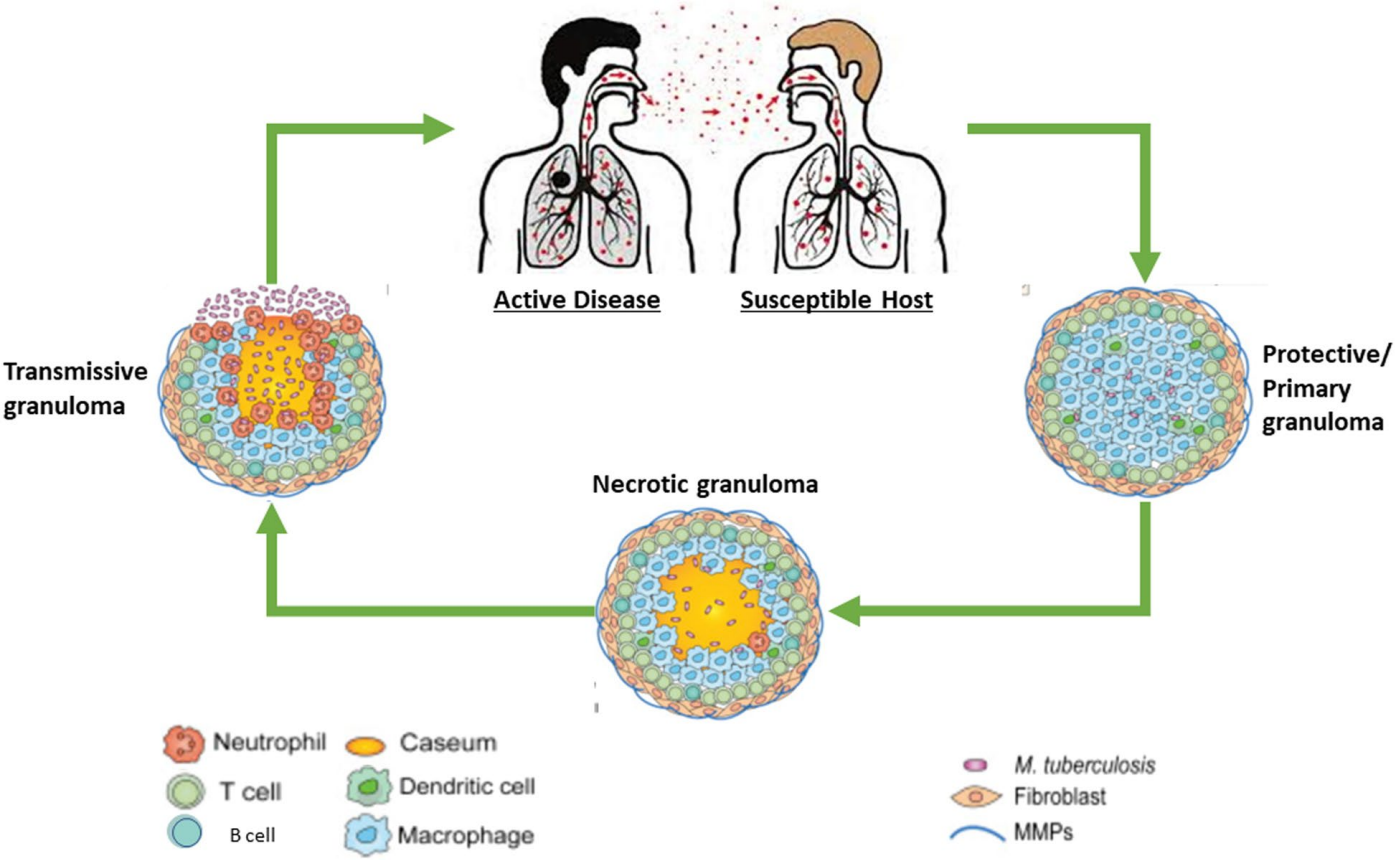
Description of TB transmission

The human lung is a complex organ located in the thoracic cavity that plays a vital role in respiration and gas exchange. The lung is composed of various structural components, including the trachea, bronchi, bronchioles, and alveoli [17]. The trachea is the main airway that connects the mouth and nose to the lungs, while the bronchi and bronchioles branch from the trachea and transport air to the alveoli [13, 17]. The alveoli are the site of gas exchange within the lungs, where oxygen is taken up by the bloodstream, and carbon dioxide is released from the body. The alveoli are lined with epithelial cells and surrounded by a network of blood vessels, enabling efficient gas exchange [7]. Understanding the anatomy of the lung is essential for the diagnosis and treatment of TB [10], as it enables clinicians to identify the site of infection and assess the extent of lung damage. Additionally, pathological analysis of lung tissues can provide valuable information about the progress of *Mtb* infection and help guide treatment decisions. Refer to Fig. 1 for graphical presentation of TB transmission.

In the early 2000s, the explosion of multimedia use over the internet saw computer vision growing from the research area to a widely accepted technology with the possibility to improve the standards of living and drastically increase productivity [2]. Not long after its initial breakthrough was achieved by [15], with their AlexNet Network, computer vision began making its way into our daily lives. Computer Vision is a study of enabling computers to understand and interpret visual information from static images and video sequences [2]. In computer vision, the majority of tasks relate to the process of procuring information for event and feature extraction. The problem-solving methods used in computer vision are dependent on the nature of the data to be analysed and the application domain. At the core of computer vision is image processing - a method to perform operations on an image, to get an enhanced image, or to extract useful information from it. It is a type of signal processing in which the input is an image, and the output may be an image or characteristics/features associated with that image [32]. Essentially, image processing consists of the following: 1) importing an image using an image acquisition tool; 2) analysing and manipulating the image; and 3) an output in which the result can be an altered image or report, based on the analysed image [36]. Image processing has been in used in medical research since the early 1970s, when image analysis techniques were developed to assist in the diagnosis of medical conditions [6]. Over the years, advances in technology and computing power have led to the development of increasingly sophisticated image processing techniques [1].

One early application of image processing in medical research was the development of tomography, which uses X-rays to create 3D images of the body [1]. This technique was first introduced in the early 1970s and has since become a standard tool in medical imaging [6]. In the 1980s and 1990s, the advent of digital imaging and the development of computer algorithms for image analysis led to further advancements in medical image processing [6, 27]. Computer-aided diagnosis (CAD) systems were developed to assist in the interpretation of medical images [36]). These systems use algorithms to analyse large volumes of data and identify patterns that may be indicative of disease. In recent years, machine learning algorithms have become increasingly popular in medical image processing, allowing researchers to analyse large datasets and identify complex patterns that may be missed by traditional image analysis techniques [6, 36]. Deep learning algorithms have shown great promise in medical research, with applications in areas such as tumour detection, diagnosis, and treatment [24]. Overall, image processing has become an essential tool in medical research, providing researchers with powerful tools to assist in the diagnosis, treatment, and management of a wide range of medical conditions. As technology continues to advance, it is expected that image processing will continue to play an increasingly important role in medical research and healthcare [24, 36].

Deep learning (DL) is an example of the machine learning paradigm of feature learning [18, 24]. Iteratively, it improves upon learned representations of the underlying data with the goal of maximally attaining class separability [4, 18]. Deep learning has had a tremendous impact on various fields of technology in the last few years and recent advances helped identify, classify, and quantify patterns in medical images [5]. Before deep learning, there were several other machine learning technologies that were utilised for various tasks. One of the earliest and most widely used technologies was decision trees [4, 20], which involve splitting data into partitions bases on certain conditions [20]. Another technology that preceded DL was artificial neural networks (ANNs), which were first theorised in the 1940s and later realised in the 1960s [16]. ANNs are like DL in that they involve layers of nodes, but they typically have far fewer layers than deep neural networks [16, 38]. Other computer vision technologies that preceded or led to DL include support vector machines (SVMs), which are particularly useful for classification problems [21], and nearest neighbours (KNN), which involve finding the most similar data points to a given input [21, 25]. Boosting and bagging algorithms are also widely used techniques that have been employed for a variety of tasks [9]. While these technologies are still widely used today, DL has quickly become the dominant approach for many tasks due to its ability to handle large amounts of complex and unstructured data [24, 26]. Numerous review papers have been published on computer vision and related techniques. However, published reviews go out-of-date. Hence, writing an updated review on computer vision is important for highlighting contemporary research directions and state of the art techniques, that can inform well-contextualised and effective future research in this space.

## Methods and materials

This section introduces rules, methods and search criteria used to retrieve relevant materials for the study. To summarise the article selection process and results, we followed the Preferred Reporting Items for Systematic Reviews and Meta-Analyses (PRISMA) reporting standard which is a systematic review protocol that describes the rationale, hypothesis, and planned methods of the review [19]. PRISMA primarily focuses on the reporting of reviews evaluating the effects of interventions but can also be used as a basis for reporting systematic reviews with objectives other than evaluating interventions [19]. We explored various terms used in the literature to understand the trend in using computer vision applications in medical imaging. Our primary focus was on publications on digitised human lung tissue for the detection and/or analysis of *Mtb* or TB pathology, then on the studies of human lung imaged ex-vivo.

In search for the most relevant papers, we first considered the domains, which are “automatic detection of *tuberculosis* from microscopic tissue images” and “the analysis of digitised human lung tissue images to detect features of *tuberculosis*”. The following database platforms were used to retrieve articles - ACM digital link, Elsevier (Engineering Village), IEEE Xplore, PubMed, and Springer Link. To further search for relevant studies for this review, we used sensitive search strategy. The following combination of keywords and search sentences were used, “*tuberculosis* + image processing + human lung tissue”, “*tuberculosis* + deep learning + human lung tissue”, “*tuberculosis* + microscopic images + human lung tissue”, “analysis and detection of *tuberculosis* from microscopic images”, “image processing techniques for *tuberculosis* analysis or detection”. The first author retrieved the literature and extracted relevant papers peculiar to the domains. To settle on the most relevant papers, the second and third authors double-checked the selections.

**Fig. 2:**
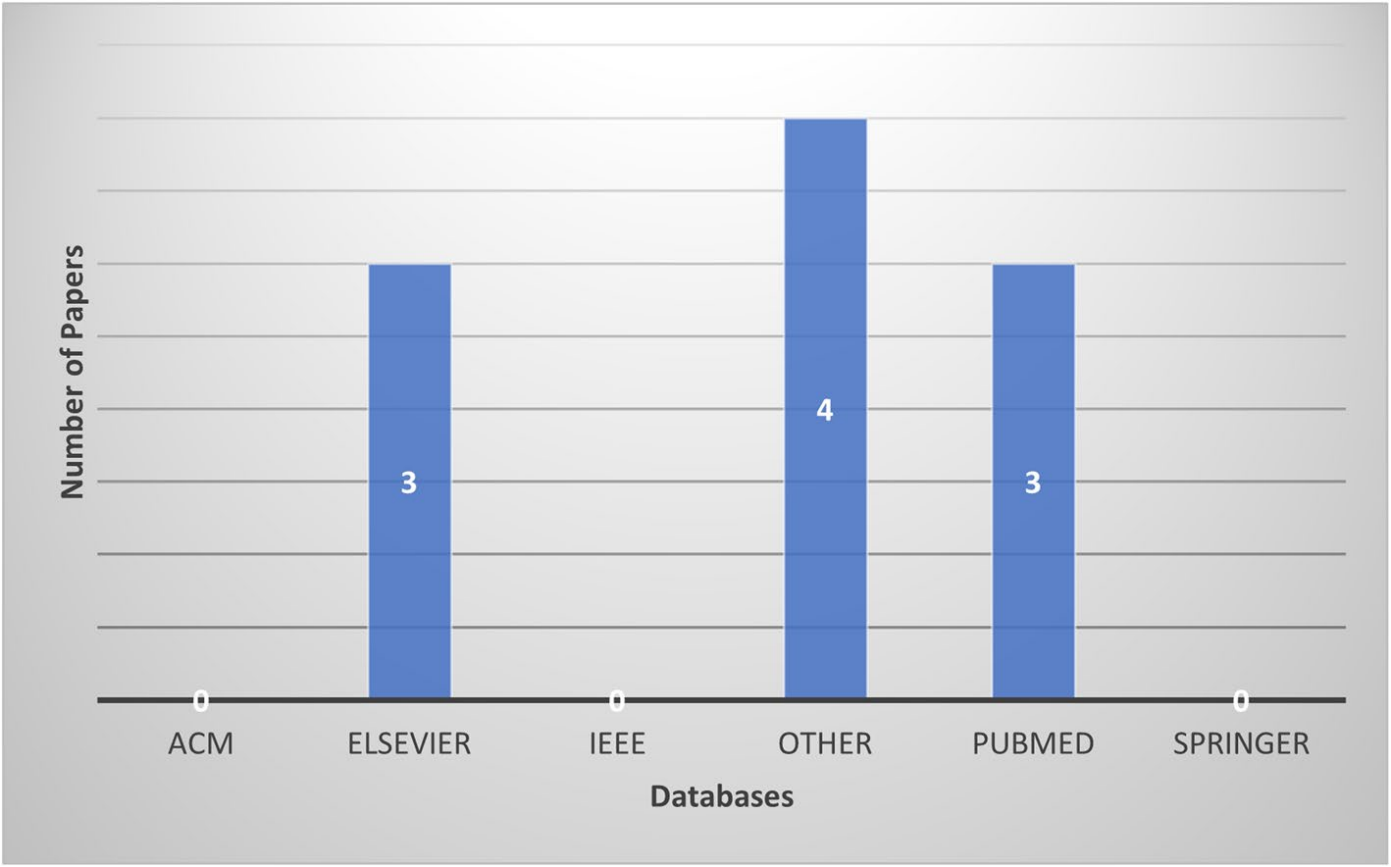
Database based distribution of this review paper

Year of publication and title of all retrieved articles were captured into an Excel spreadsheet where duplicated titles were identified and removed. For each database used to retrieve the articles, two sets of folders were created; unrelated abstracts were identified and moved to the secondary folder of the respective database. Publications on computer-aided, image processing, and deep learning techniques proposed for automated or semi-automated detection or analysis of TB using digitised human lung tissue images, and those on ex-vivo imaging of human lung tissue were considered. Articles on similar techniques implemented on different image modalities such as in-vivo Chest X-ray, Chest CT and MRI or digitised image of sputum smear were excluded. A total of 165 articles were retrieved, and 21 were found to be duplicates due to the various searches that we performed from different databases. Thus, the total number of retrieved articles was brought down to 144. After careful consideration, a total of 10 articles were selected for this review (Fig. 2 provides the breakdown). It important to note that the low number of publications is due to the lack of TB or lung studies conducted using human lung tissue.

The selected papers were segregated into two categories based on digitised microscopic images of human lung tissue, and images from ex-vivo CT/micro-CT scan of human lung tissue. Two reviewers independently extracted data using a standard form on Microsoft Excel - one spreadsheet for articles that used microscopic images and the other for CT/ $\upmu$CT imaging. The following information was extracted from these articles: year of publication, geo-location, source of funding, study design, method of generating digital images, segmentation and classification techniques, computer-aided models or development, dataset used, model performance and validation, sample preparation and mounting, limitations and risk of bias, research gaps, and outcomes.

## Research questions

The main objectives of this review are addressed through the following research questions: (1) What types of image processing techniques are necessary to detect or analyse *Mtb* or TB pathology on digitised human lung tissue images? (2) What types of deep learning models are used in the detection and/or analysis of *Mtb* or TB pathology from digitised human lung tissue images? (3) What image datasets are available in the study of *Mtb* or TB pathology using digitise human lung tissue images? (4) What is the current state of *Mtb* detection accuracy and TB pathology analysis on digitise human lung tissue images?

## Results

### Study selection

**Fig. 3:**
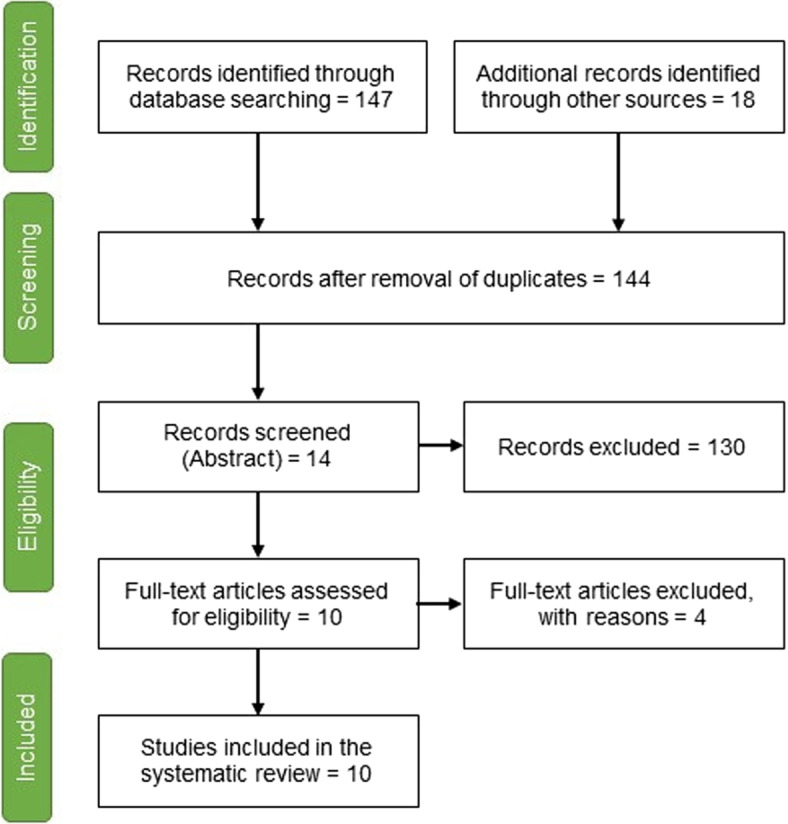
Article selection flow diagram [19]

As depicted in Fig. 3, a total of 165 articles were retrieved from various databases; of which 144 remained after de-duplication, and 10 articles were included after full text assessment, of which 5 used digitised images of histology slides of human tissue (predominantly lung), and the remaining 5 articles used images from human lung CT scanned ex-vivo.

Of the 10 selected articles for this review, 5 used computer vision architectures to assist in reducing the time it takes Pathologists to diagnose TB and improve detection or diagnostic accuracy by developing automated or semi-automated models as either an aid to the Pathologist or a standalone diagnostic system. Two of the five articles were published in 2022 and were conducted in Romania and Japan, while the remaining three were published in 2018, 2020 and 2021 respectively from China, United States of America (USA), and the Republic of South Africa (RSA). The remaining five studies analysed human lung structures using CT images generated from the ex-vivo scan of human lung samples. Two of the studies were conducted in RSA and Belgium and published in 2021. The other three were conducted in United Kingdom (UK), Canada, and France and were respectively published in 2019, 2020 and 2022. Yang et al. [35] conducted a retrospective study and, [29] conducted a cross-sectional study while the others did not stipulate which study design was followed.

### Summary of studies

This subsection briefly introduces the 10 articles that were reviewed, highlighting their focus areas and reported results. Subsequent sections will build on this introduction by firstly presenting the observed common themes among the studies ranging from methods used to generate digitized images to metrics used for performance evaluation, then highlighting the unexplored research gaps.

The paper by [22] presents an artificial intelligence (AI) based approach for the screening and detection of *mycobacteria* in whole-slide images of human tissue samples. The authors used a deep learning algorithm to classify tissue samples as mycobacterial or non-mycobacterial. The algorithm was trained on a dataset of 3,096 tissue samples, achieving an accuracy of 96.4%. The authors further evaluated the algorithm on an additional dataset of 795 tissue samples, achieving an accuracy of 86.2%. Xian et al. [33] paper presents a novel approach to the automatic detection of *MTB* using AI. The authors propose a deep learning model for *MTB* detection that involves a combination of convolutional neural network (CNN) and long short-term memory (LSTM) networks. The model achieved an accuracy of 94.62%. The paper by [35] proposes a CNN-based active learning framework for the identification of *mycobacteria* in digitalised ZN-stained human tissue samples. The framework combines the strengths of CNN-based deep learning with active learning, which involves selecting informative samples for annotation to improve model performance. The framework was evaluated and achieved a mean average precision (mAP) of up to 96.41%. The paper also includes a comparison of the proposed approach with other machine learning-based methods for mycobacterial detection. Zurac et al. [39] present a new AI-based method for identifying *Mtb* in ZN-stained human tissue samples. The authors developed a convolutional neural network model that was trained on a dataset of 1,880 ZN-stained tissue samples, achieving an accuracy of 96.3%. The paper by [37] presents a deep-learning detection approach for the identification of *mycobacteria* in human pathology specimens, which the authors show to be more sensitive in early diagnosis of PTB when compared with standard bacteriology tests. The model was trained and validated using a dataset of 1,123 pathology specimens from patients with suspected PTB, achieving a sensitivity of 94% and a specificity of 92%. Furthermore, the results of the deep learning aided detection method were compared with bacteriology tests in a real-world clinical setting, demonstrating that the deep-learning approach was significantly more sensitive in early detection of PTB.

Xian et al. [33] presented a multiscale X-ray phase contrast tomography dataset of a whole human left lung, which they acquired using the X-ray microscopy beamline of the Canadian Light Source synchrotron. The dataset contains high-resolution images of both the microvasculature and the alveolar structure of the lung, enabling investigations into the lungs’ respiratory function and diseases such as chronic obstructive pulmonary disease (COPD) and lung cancer. The authors described the techniques used to acquire and process the data, as well as the features of the dataset that make it useful for researchers in the field. They also provided examples of how the dataset could be used for lung imaging studies, such as generating 3D reconstructions of the lung’s architecture and analysing the distribution of the air spaces and blood vessels. Overall, the dataset provides a valuable resource for investigating human lung structure and function at a range of scales. Wells et al. [30] used $\upmu$CT to analyse the granulomas (masses of immune cells) that form in human lung tissue because of *Mtb* infection. The authors noted that previous research on TB granulomas has primarily used two-dimensional (2D) imaging techniques, which may not accurately represent the 3D structure and heterogeneity of the granulomas. Using $\upmu$CT, the authors were able to generate high-resolution, 3D reconstructions of the granulomas in lung tissue samples from human TB patients. They found that the granulomas varied significantly in size, shape, and overall morphology, with some containing dense clusters of immune cells while others contained large area of necrotic tissue. Furthermore, the authors observed that the granulomas showed evidence of reorganization over time, with changes in size and shape occurring even within a single sample.

In their publication, [14] describe a new imaging technique that combines two types of X-rays to create highly detailed 3D images of biological tissue samples, enabling researchers to study the structure of tissues in unprecedented detail. This approach, called X-ray micro-computed 3D X-ray histology ($\upmu$CT histology), uses one of the X-ray to create a 3D map of the tissue’s microstructure and another type to highlight specific features, such as blood vessels or bone. On the other hand, [28] describe a study that investigated the effects of aging on the number and size of small airways in the lungs. The authors examined unused donor lungs from individuals across a range of ages and used multiple imaging and analytical techniques to characterise the lungs and assess the number and size of small airways. They found that the number of small airways decreased with increasing age, with a more rapid decline after the age of 65. The authors also found that the effect of small airways decreasing in size with age was more pronounced in female compared to male donors. Lastly, [8] describe a study that used multiresolution CT to assess the 3D structure of the human lung. The researchers used a series of images processing techniques and stereological methods to analyse the lung structure at different levels of resolution, from the whole lung down to the individual alveoli. The authors found that the lung structure varied greatly between individuals, and that there were significant differences between healthy lungs and those affected by emphysema.

## Methods of generating digitised images

### Samples collection

All tissue specimens from the five studies on machine learning methods were fixed, paraffin-embedded, sectioned, and stained by acid-fast stain following the standard protocol. Some were surgical pathology samples and others were cytology cell block or autopsy samples. As summarised on Table 1, a total of 246 cases were collected from the Department of Pathology, Peking University First Hospital [34]. Yang et al. [35] used 167 samples collected by bronchoscopy from the Pathology department files at Cedars-Sinai Medical Center [35]. Pantanowitz et al. [22] used 556 whole slide images (WSIs) collected from the University of Pittsburgh Medical Center (archival cases from 2016 to 2019), and the Wan Fang Hospital, Taipei Medical University - Taiwan, while [37] collected samples from 2 autopsy cases of pulmonary TB, and 40 biopsy cases of undetected acid-fast *bacilli* (AFB) for training and validation, and 510 WSIs of ZN-stained slides (110 positive and 400 negative) from the 2010 to 2022 archival cases of the Department of Pathology of Colentina University Hospital were used by [39].

**Table 1:** Studies of automated detection of AFB on ZN-stains on human tissue

Studies on Tissue		Xiong et al.	Yang et al.	Pantanowitz et al.	Zaizen et al.	Zurac et al.
Year		2018	2020	2021	2022	2022
Total Cases		246	167	556	84	570
Training Set (WSIs)	Pos.	30	6	47	2	110
	Neg.	15	27	371	40	400
Test Set (WSIs)		201	134	138	42	60
Patches	Pos.	96,530	18,246	5678	506	263,000
	Neg.	2,510,307	18,246	1,111,918	*	700,000,000
	Pixels	32 x 32	256 x 256	32 x 32	*	64 x 64

* Data not available

The remaining five studies scanned human lung tissue ex-vivo using CT and/or $\upmu$CT scans (refer to Table 2). Xian et al. [33] imaged ex-vivo an entire human left lung from a 94-year-old woman using the fourth-generation synchrotron source at the European Synchrotron Radiation Facility, while [30] analysed lung specimens obtained from 17 patients who underwent either a lobectomy or pneumonectomy procedure for the removal of irreversible damaged lobes or lungs; and the other two studies collected non-transplantable lungs from organ donors [8, 28]. Verleden et al. [28] collected 32 lungs from patients aged between 16 - 83 years from the University Hospital Leuven, while [8] used 13 donor lungs (age range 25 - 27 years) consisting of 3 left and 10 right lungs from individuals with no evidence of respiratory disease. These lungs were collected through the University of Pennylvania (n = 7), University of Michigan (n = 3), and Katholieke Universiteit Leuven (n = 3). The last study used two human lung tissue samples - one control sample from a macroscopically normal lung and a diagnostic surgical lung biopsy sample that was confirmed to have a typical interstitial pneumonia pattern by two independent pulmonary pathologists [14].

**Table 2:** Studies on ex-vivo imaging of human lung tissue

Studies	Katsamenis et al.	Dragos et al.	Verleden et al.	Wells et al.	Xian et al.
Year	2019	2020	2021	2021	2022
Specimen	2 biopsies	13 lungs	32 lungs	17 samples	1 left lung
Source	Surgical	Explanted lung specimens from organ donors	Explanted lung specimens from organ donors	Surgical	PM
Patients Age	*	25-77 yrs	16-83 yrs	*	94 yrs
Fixation	FFPE	Inflated with air - 30 cm	Inflated with water - 30cm pressure	10% formalin	4% formalin and inflated with water - 30cm pressure
Contrasting	*	Frozen in liquid nitrogen - 1h	Frozen in liquid nitrogen	Iodine - 2.5% Lugol’s solution	Absorption contrast

* Data not available

### Samples preparation and mounting

Different methods and techniques were used in sample preparation and mounting for the selected studies on human lung specimens. Samples from two studies [28, 33] were inflated using water pressure, while another study [8] inflated the samples with air at a specific pressure and froze them rapidly. The explanted lungs from [14] specimens were cooled, banged, and transported while inflated on ice, and then frozen at a constant pressure; and the ones from [30] were fixed in formalin and contrast stained with iodine. In the study from [33], a formalin solution was instilled into the sample through the trachea and maintained the inflated configuration by ligaturing the trachea. The specimens were then immersed in formalin solution and subsequently dehydrated with ethanol. The use ethanol provided contrast for soft tissues due to its lower density compared to water.

Furthermore, different methods were used for mounting the samples. One study [14] used acrylic polymer cylinders and polyethylene foam, another used falcon tubes and floral foam [30], and another used a PET jar with agar powder to secure the specimen [33]. The mounting procedures involved degassing the agar-ethanol mixture to minimize the appearance of microbubbles [33]. In some cases, cryo-stages were used to prevent sample shrinkage [8, 28].

### Imaging and segmentation

Slides from [34] study were scanned using KF-Pro-005 Digital Section Scanner (Ningbo Jiangfeng Bio-information Technology Co., Ltd., Ningbo, China). Zaizen et al. [37], used Motic EasyScan (Motic, Hong Kong, China). Different versions of the Aperio scanner (Leica Biosystems) were used on 3 studies: [35] used the AT version to digitalise 14 slides into WSIs, [22] used the AT2 version in addition to Hamamatsu Nanozoomer XR, and [39] used GT450 in addition to a manual method consisting of a Microvisioneer mounted with a camera Basler Ace 3.2MP and a Sony IMX265 Sensor. Different custom computer vision architectures were used in these studies, coupled with several image augmentation techniques. With exception of the [22] study, image augmentation techniques such as rotation, position shifting, mirroring, saturation, contrasts, etc. [27], were applied on the WSIs of the other 4 studies, which eventually increased the set of positive patches (patches with *bacilli*). These patches were mostly separated in three groups for: training, analytical validation, and testing. To label the *bacilli* in the positive patches, [34] used the ASAP software, [37] made use of HALO software, and a web-based application (aetherSlide) was used by [22], while [35] and [39] did not explicitly state which platform was used for patch labelling in their respective studies. Detailed number of patches are depicted on Table 1.

All X-ray imaging experiments from [33] study were carried out at the European Synchrotron Radiation Facility (ESRF) bending magnet beamline BM05. Without any additional X-ray optics, the produced polychromatic synchrotron beam passed through a set of filters, then directly used for imaging. An adjustable visible-light imaging optics effectively controlled the voxel size. Wells et al. [30] scanned the samples over 360o with a varying voltage between 8 and 160 kV, current of 180 - 400 mA, and scanning time ranged from 2,700 to 3,000 seconds using a General Electric Phoenix v|tome|xL240 system for $\upmu$CT imaging (2,024 X 2,024-pixel image, 16-bit depth) with a resolution range of 12.0-80.0 mm. Verleden et al. [28] on the other hand used two different scanners for imaging - a high resolution $\upmu$CT scanner (Skyscan 1172, Bruker; Kontich, Belgium) was used for the frozen samples at -30oC (0.5 rotation step, 3 frames per rotation). The scanner was operated at 40 kV and 226 $\upmu$A. The other six frozen, inflated right lungs surrounded by dry ice were scanned using an in-house developed $\upmu$CT system HECTOR operated at 150 $\upmu$m voxel size, 80 kV voltage, and target current of 208 $\upmu$A.

To determine the total volume of the specimens, one study used CT imaging on frozen human lung samples [8]. The CT parameters used were 120 kV, 250 mA, and a 1-second exposure time [8]. For the imaging of frozen systematic Uniform Random (SUR) samples, a $\upmu$CT scanner with parameters including 40kV, 350 uA, molybdenum target, 500-ms exposure time, and a gain of 32 dB was employed [8]. Image registration was performed using a custom program developed in MATLAB, aligning the ex-vivo specimen CT scan with photographs of the cut specimen slices. To minimize interference from the cassette, another study carefully removed the FFPE lung tissue blocks from the histology cassettes [14]. A $\upmu$CT imaging was then conducted with parameters including a molybdenum target, an accelerated voltage of 55 peak kV, and no X-ray prefiltration [14]. The filament current was set at 125 mA, and the source-to-object and source-to-detector distances were adjusted to 42.1 mm and 992.0 mm, respectively, resulting in an isotropic voxel size of 8.48 mm. A total of 3501 projections were collected over a 360-degree angular range, and four frames were averaged per projection to enhance the signal-to-noise ratio.

### Reconstruction and analysis of 3D images

As specified by [29, 33], the data processing of X-ray projections involve three main stages: pre-reconstruction, reconstruction and post-reconstruction. Further, the ring artifacts from the detectors are addresses through two correction steps: prior to reconstruction, the mean of the projections is subtracted from them to eliminate rings with constant intensity, and after reconstruction residual inhomogeneous intensity rings are removed using a polar transform combined with a linear motion blurring filter as described in [33]. The resulting volumes are then converted to a 16-bit format and later further binned to create datasets, as mentioned in [1]. Table 3 summarises the tools used in ex-vivo imaging.

**Table 3:** Tools used on ex-vivo Imaging of human lung tissue studies

Studies	Year	Mounting	Scanner used	Reconstruction	Segmentation	Classification and Analysis
Katsamenis et al.	2019	Thin-walled stackable acrylic polymer cylinders and stabilized with polyethylene foam	Molybdenum target	CT Pro; Nikon	Fiji/ImageJ (ver. 1.51n)	Amira ver. 6.1.1
Dragos et al.	2020	Cryomicro-CT stage	XT H 225ST; Nikon	*	*	*
Verleden et al.	2021	Cryo-stage	Skyscan 1172 and HECTOR	Bruker Nrecon (Ver. 1.7.4.2) and Octopus Reconstruction	ITK-SNAP (ver. 3.7.0)	NEURON Studio, VIDA Vision, and Bruker CTAns
Wells et al.	2021	50 ml falcon tubes with floral foam	General Electric Phoenix v|tome|xL240	Bruker Nrecon	*	*
Xian et al.	2022	Agar power and the polyethylene terephthalate (PET) jar	European Synchrontron Radiation Facility (ESRF)	PyHST2	*	*

*Data not provided

Studies [28, 30] used the Bruker Nrecon software for scan reconstruction, with [28] specifying version 1.7.4.2. In addition to Nrecon, [28] employed the Octopus Reconstruction (XRE) for 3D volume reconstruction with voxel size of 150 $\upmu$m. The CT datasets from [28] were loaded into ITK-SNAP for semi-automated 3D airway segmentation. This study also used NEURON studio to detect and classify airways from the segmented 3D airway model. They also used the VIDA vision software to analyse CT images, assessing emphysema and central airways up to the tenth generation. Furthermore, [28] used the Bruker CTAns software version 1.18 to determine tissue volume and surface density.

Following micro-CT acquisition, [14] reconstructed the data into 32-bit raw volume files using Nikon’s CT reconstruction software (CTPro version V5.1.6054.18526). They imported the reconstructed raw data volumes into Fiji/ImageJ software and applied a 3D median filter and a 2D unsharp mask to enhance image quality. The resulting 16-bit CT volumes were calibrated against a custom-made contrast phantom containing standard histology-grade paraffin wax. Grey values corresponding to air and wax were identified in both the sample and the phantom. Although [7] did not specify their reconstruction methods, they did provide information about the parameters used, including calculations of total lung and parenchymal volumes based on semi-automatic image segmentation, excluding the main stem bronchus and surrounding connective tissue.

## Computer-aided models (development)

The study from [34] developed a convolutional neural network (CNN) model called the TB-AI using a 45 whole slide images (WSIs) training set of which 30 were positive and 15 were negative, and a further 201 WSIs (108 positive and 93 negative) for testing. The digital WSIs were subdivided into patches measuring 32 x 32 pixels. The patches utilised for both the training and testing of the algorithm. The model examined these patches and marked them as “positive” when the probability score exceeded 0.5. If at least one patch in a WSI was labeled as “positive”, the entire WSI was categorised as such. This approach, described in [34], is entirely automated and doesn’t involve human examination for classifying WSIs.

In 2020, [35] introduced an AI-assisted diagnostic method that combines a CNN model (Inception-V3) for patch-based classification and a Logistic Regression model for the overall WSI classification. Patches of 256 x 256 pixels were extracted from WSIs and used to train the CNN model to detect AFB. Subsequently, a semi-supervised active learning framework was employed to retrain the model using additional patches from 19 negative WSIs. The retrained CNN model then classified patches as “positives” or “negative” from a set of 134 WSIs (46 positive and 88 negative). The results of the CNN classification were used by the LR model to classify the entire slide as either “positive” or “negative.” The model generated a score heat map indicating the likelihood of AFB, superimposed onto the WSI. Pathologists would then examine the regions within this heat map to confirm the WSI’s positivity.

Using two deep CNN models, [22] developed an algorithm to screen for *mycobacteria* within digitised tissue section on slides. This dual-model approach aimed to filter out false-positive predictions, with the first model prioritising sensitivity and the second focusing on specificity. The study from [22], developped an AI-Assisted screening method that presented a gallery of patches along with their corresponding probability scores and the full WSI, allowing for an in-context evaluation of suspicious patches. A web-based application called “aetherSlide” was employed to annonate WSIs and generate training patches. The authors of [37], developed an algorithm for AFB detection in tissue sections using a pre-trained HALO AI CNN, referred to as an AI-supported diagnosing method. Each patch identified as potentially positive by the algorithm underwent evaluation by a consensus of six pathologists.

Incorporating a considerably larger dataset, [39] developed an AI-based automated method of identifying *mycobacteria* in digitised tissue images using the Deep Neural Network (DNN) model. This model was developed by customising the RegNetX4 architecture, a Deep CNN known for its performance, simplicity, and speed [23]. This AI-assisted diagnostic method, akin to [22]’s platform design, presented a list of patches ordered by their probability scores, albeit with a larger dataset and an active learning approach that enhanced performance metrics, particularly for challenging cases.

### Model performance and validation

The CNN model presented in the study by [34] underwent two rounds of testing. During the first evaluation, instances identified as false positives and false negatives by human evaluator were subjected to re-assessment by two pathologists. In this initial test, seven cases were initially overlooked by the human evaluator, while six cases were deemed unsuitable due to poor scan quality. When compared against the double-confirmed diagnosis by Pathologists, TB-AI model demonstrated an impressive sensitivity of 97.94%, and specificity of 83.65%. The models from [35], as discussed in the same paper, were subjected to validation using a distinct set of patches and achieved F1 scores of 99.03% and 98.75%. Furthermore, at the WSI level, the performance metrics for their pipeline were - 87.13% for sensitivity, 87.62% for specificity, and a F1 of 80.18%.

For the [22] model, clinical validation was carried out on 138 slides. This validation process involved blind evaluations conducted by two pathologists with varying levels of expertise. It encompassed classical microscopic slide examination, evaluation of the WSIs, and AI-assisted assessments. The model’s performance was reflected in an accuracy of 84.6%, sensitivity of 64.8%, and a specificity of 95.1%. In the case of the [37] method, a clinical test involved 42 cases, encompassing patients diagnosed with *mycobacteriosis* via bacteriological tests from bronchoscopy material or those who developed *mycobacteriosis* during follow-up. This method achieved performance metrics of 86% sensitivity and 100% specificity. In the [22] method, the Pathologists evaluated a gallery of patches in reverse order of their probability scores in relation to the WSI. In contrast, the [35] method involved pathologists assessing a score heatmap overlaid on the WSI, facilitating the evaluation of suspicious areas within the context of specific histopathological lesions. However, [37] did not provide a precise description of how the Pathologist utilised the platform for diagnosis; instead, they mentioned that each potentially positive patch was examined by six Pathologists. Another study by [39] involved testing their model with 60 ZN slides and subsequent internal validation on a dataset of 286,000 patches. Their AI-assisted diagnosis model achieved an accuracy of 98.33%, a sensitivity of 95.65%, and specificity of 100%. For detailed statistical measures of the models’ performance on whole slide images, please refer to Table 4.

**Table 4:** Statistical measures of the models’ performance on whole slide images

Studies	Year	Sensitivity %	Specificity %	Precision	Accuracy %	F1
Xiong et al.	2018	97.94	83.65	*	*	*
Yang et al.	2020	87.13	87.62	*	87	*
Pantanowitz et al.	2021	64.8	95.1	*	84.6	*
Zaizen et al.	2022	86	100	*	*	*
Zurac et al.	2022	95.65	100	1	98.33	0.9778

*Data not provided

## Limitations and risk of bias

All the studies included in this review have limitations. First a limited number of human lung tissue specimens were examined from patients with different medical histories and/or treatments. This also prevented the correction for multiple testing and analysis of differences in lung structure between - for instance men and women [33]. Second, the results from studies that focuses on diseased - TB patients [30] and pneumonia [14] may not represent a moderate manifestation of the disease. Third, studies that used specimens from organ donors could not know for sure whether the donors had a normal lung function because the baseline spirometry and occupational exposure was not provided due to the anonymity of organ donation [8, 28]. Despite the many advantages of $\upmu$CT for detailed assessment of the lung anatomy, it still cannot be performed in-vivo [8, 28, 30]. while the XPCT technique used in the study by [33] provides high-resolution images, it is also associated with radiation exposure, which remains a concern, particularly in longitudinal studies; and the dataset used is not accompanied by clinical metadata.

Regarding the AI model by [34], it achieved excellent sensitivity in identifying most *bacilli* but relatively low specificity, leading to a substantial number of false-positive results. Common machine learning practices prohibit using the test dataset twice to maintain model objectivity. However, [34] retested the dataset after identifying label errors in the first run. Some of the limitations of ther study include the relatively small number of cases in the dataset, which may limit color variability representation in the input space. Furthemore, pathologists must verify samples labeled as “positive” by TB-AI and review those labeled “negative” to ensure the digital slides meet quality standards. In [35]’s model, the generation of a score heat map overlaid on WSIs guided pathologists in analysing probable positive patched. However, the model’s low specificity of 87.62% indicates a high number of patches falsefy labeled as negative, necessitating pathologists to examina a large number of patches suggested as positive. Consequently, the time and effort required to analyse the results might surpass that of classical microscopic examination. additionally, the dataset’s diversity is limited. An ideal dataset should encompass slides or WSIs from diverse geographical locations [37, 39]. The main limitations of [39]’s study pertain to the dataset’s dimensions and diversity, as well as the methodology of clinical testing, which involved the researchers who developed the model in the validation process. To address these limitations, it is imperative to validate the algorithm using pathologists from entirely independent institutions across numerous countries, involving an international patient cohort.

The AI-supported pathology from [37] has proven to be more sensitive than bacteriological tests for detecting AFB in samples collected through bronchoscopy. However, this was not achieved without limitations; such that the study used WSI produced by a single scanner only and deep learning in one model, only autopsy cases of TB were used as AFB positive training data, and another potential limitation is that the proposed approach may detect other acid-fast *bacilli* species that are not *Mycobacterium tuberculosis*, potentially leading to false positives (approximately 200-500 false positives for every true positive). Furthermore, the proposed approaches [34, 35, 37] rely on the quality of the input pathology specimens. If the specimens are of poor quality or have artifacts due to poor staining, they may produce unreliable results.

## Research gaps

None of the studies on the WSIs explicitly identify any research gap; however, it is possible to infer a few potential research gaps based on the limitations and future research directions discussed in these papers. For instance, [22] mentioned that the algorithm’s performance may be impacted by the quality of tissue samples or the presence of artifacts. Therefore, future research may focus on developing techniques to improve the quality of digital images for analysis or to handle the presence of artifacts better. [39] acknowledged that the proposed approach was designed to detect only acid-fast *bacilli* and may, therefore, miss the detection of non-acid-fast organisms. Further research may explore the feasibility of developing an AI-based approach that can detect a range of organisms, potentially using a combination of staining techniques. [35] mentioned that the accuracy of their method may be impacted by the quality of the stained samples; thus, future research may focus on developing techniques to improve the quality of digital images for analysis which could potentially improve the accuracy of the proposed methods.

The dataset used to train the algorithms may not be comprehensive or was relatively small [22, 34, 35], was obtained from a single institution or source [37, 39], limiting the generalisability and transferability [35] of the proposed approach. Therefore, future research may focus on increasing the size and diversity of the dataset to improve the performance and the external validity of the proposed methods; and evaluating their performance on different datasets or in different healthcare settings. Additionally, [37] acknowledged that the proposed approach had limitations identifying extrapulmonary TB, which may require further research to address. Zaizen et al. [37], and [34] also did not compare their proposed approaches with other existing AI-based TB diagnosis or detection methods, which could help determine the relative advantage of the proposed approach with current options. Zurac et al. [39], and [37] did not explicitly discuss how their proposed approaches could be integrated into clinical workflows or evaluate its potential impact on clinical decision-making. Thus, future research may investigate the practical implementation and clinical efficacy of the proposed approach and analyse the cost-effectiveness of such implementation. Pantanowitz et al. [22] did not explore the use of the proposed approach in immunosuppressed patients, who may have a greater incidence of *tuberculosis*. Therefore, future research could evaluate the potential of the proposed approach in populations with a higher risk of *tuberculosis*.

Although the study by [30] sheds new light on the complex 3D morphology of TB granulomas, the following gaps still remained - (1) the study was limited to postmortem samples which may not fully reflect the complexity and diversity of *Mtb* infection in humans, (2) the study also had a relatively small sample of TB patients; further studies are needed to confirm the findings and assess their generalisability to other TB cases, (3) the researchers did not investigate the impact of different factors such as age, gender, or co-infections, on the 3D morphology of TB granulomas. Future studies could explore these associations. (4) The study focused on pulmonary TB, studies investigating the 3D morphology of granulomas in extrapulmonary TB cases are also needed. The study by [14] does not specifically address any research gap. However, it should be noted that one of the main limitations of the traditional histology techniques is that they are limited to examining thin slices of tissue, which can be time-consuming and may miss important structural features. $\upmu$CT histology offers a promising new approach to overcome these limitations and to provide more detailed and accurate 3D imaging of tissues. While the technique has been shown to be effective in several applications, further research will be needed to fully evaluate its potential and to optimise its use of specific tissue types and research questions. The study by [28] is limited in its cross-sectional nature and the use of donor lungs, which may not fully reflect the aging process in non-diseased lungs. The authors recommend future longitudinal studies in healthy individuals to clarify the relationship between small airway loss and lung aging. Overall, this study highlights the need for continued research to better understand the effects of aging on the lung and to develop effective interventions to prevent and treat age-related lung diseases like COPD.

The study by [8] did not explicitly identify any research gaps either, as it was focused on presenting the findings of a comprehensive stereological assessment of the human lung using multiresolution computed tomography. However, some potential research gaps could be identified from the study’s findings could include: (1) future investigation of the relationship between lung structure and lung function in both healthy lungs and lungs affected by disease. (2) characterization of the changes in lung structure that occur over time in response to environmental exposures or therapeutic interventions. (3) Exploration of the potential of multiresolution computed tomography to monitor lung health and disease progression in clinical settings. Furthermore, [8] recommend that future studies of chronic lung diseases should build on the detailed stereological measurements of the alveoli, ducts, and parenchyma as reported in their study. This, they believe, will be crucial in trying to determine the exact changes in tissue pathology and microscopic lesions that cannot be detected by clinical CT. While the study by [33] provides a rich and valuable dataset that can facilitate research in lung biology and disease, there are still some research gaps: (1) the dataset is from only one left lung, and thus may not be generalised to all lungs. Future studies should include more populations to explore inter-individual variability, (2) the authors do not provide any clinical applications of the data, such as the identification on pathological features of the lung disease or identification of biomarkers for diagnosis or predicting disease progression, (3) the presented analysis did not utilise image analysis techniques like deep learning for automatic feature identification, where additional structures or features could potentially be explored. These gaps highlight the need for further research to complement these studies and further understand lung biology and disease.

## Discussion

Overall, the study by [33] presents a novel method for acquiring high-resolution 3D images of human lung structure and topology, which can benefit research in lung biology and disease. The findings from the study by [30] suggest that the heterogeneity of TB granulomas may have important implications for the progression and treatment of Mtb infection and highlight the potential value of 3D imaging techniques such as $\upmu$CT in the study of infectious diseases. Their findings, [30], open new avenues for research on TB morphology but further studies are needed to address the stipulated research gaps and to deepen our understanding of TB granulomas and the development of novel treatment approaches. The technique presented by [14] offers a powerful tool for understanding the complex structure of biological tissues and for studying the effects of diseases or therapies on tissue structure, while the findings from [28] study highlight the importance of better understanding the mechanisms of age-related lung disease and potential targets for intervention. The study by [28] further suggests that small airway loss contributes to the physiological aging of the lung and may contribute to age-related lung disease such as COPD; while the study by [8] provides a comprehensive framework for understanding the complex and intricate structure of the human lung and may lead to new insights into lung disease and their treatment.

The proposed machine learning methods have significant potential for use in clinical diagnosis and management of TB, as they provide a fast, accurate [22, 34], and efficient [35, 39] means of screening and detecting *mycobacteria* in digitised human tissue samples, enabling early detection and treatment initiation. The proposed approach by [37] has the potential to improve the sensitivity and specificity of PTB diagnosis and has significant implications for public health initiatives aimed at preventing the spread of TB. The approach by [39] may also have applications in the detection of other infectious agents, making it a versatile tool for digital pathology. Despite the possibility of recall bias caused by the 7-days minimum washout period used between review methods during the clinical validation, the study by [22], reported a successful development and clinical validation of an AI-based digital pathology system to screen for AFBs in anatomical pathology material. It is evident that for most (if not all) of these studies, the authors developed their own image dataset using the pathological material at their disposal. Hence is highly important for future research to focus on developing standard sample preparation documents and datasets of digitised human lung tissue images from different parts of the world. Such benchmark repositories will enable object comparative research on computational methods that enhance automated TB analysis and diagnosis. The findings of this study indicate that 3D structural lung tissue details are useful for analysing both the severity and progression of TB. It is therefore recommended that where possible such datasets prioritise 3D representations, patient age information, and repeated samples from the same patient over time.

In response to the research questions: (1) it is evident that the use of a combination of image processing techniques such as pre-processing techniques, segmentation techniques, feature extraction techniques, and classification techniques is critical in achieving accurate detection and analysis of *Mtb* or TB pathology on digitised human lung tissue images; (2) several types of deep learning models such as convolutional neural network (CNNs), recurrent neural networks (RNNs), and transfer learning have been used for the detection and analysis of *Mtb* or TB pathology from digitised human lung tissue images. However, the choice of model depends on the specific task and the available data. (3) Several image datasets are available for studying *Mtb* or TB pathology, but most of these datasets contain either digitised chest X-rays or images of digitised images of ZN-stained sputum smear slides. However, it is important to note that each dataset has its own characteristics and limitations, and researchers should carefully consider the dataset that is most appropriate for their specific research question and application. Hence, the authors of the selected studies each built their own dataset. Lastly, (3) the current state of *Mtb* detection accuracy and TB pathology analysis on digitised human lung tissue images varies depending on the specific task, dataset, and evaluation metric used. However, overall, there has been significant progress in the development of image processing and machine learning algorithms for the detection and analysis of *Mtb* and TB pathology from digitised human lung tissue images.

## Conclusion

A number of studies have reported high accuracy in the detection of *Mtb* in ZN-stained human tissue slides using image processing and machine learning methods. sensitivity and specificity of 95.65% and 100% respectively have been reported using a deep learning model [39]. Overall, the studies in this review suggest that significant progress has been made in the development of image processing and machine learning algorithms for the detection and analysis of *Mtb* infection and TB pathology from digitised human lung tissue images. However, there are still challenges in developing accurate methods that can be widely applied in clinical practice.

To advance the field of computer-aided methods in diagnosing *Mtb* infection and TB pathology, a multifaceted approach is essential. First and foremost, there is a pressing need for studies that address the identified research gaps comprehensively. These studies should encompass diverse populations, including those with varying disease securities, demographic characteristics, and geographical locations. By capturing a broad spectrum of data, researchers can develop algorithms that are not only accurate but also robust and applicable across different clinical scenarios. Standardization of methodologies represents another crucial aspect to consider. While innovation drives progress, standardization ensures consistency and comparability across studies. Establishing common protocols for data acquisition, image processing, and algorithm validation can facilitate the replication and validation of findings.

In addition to standardization, interdisciplinary collaboration is vital for advancing the field. Collaboration between computer scientists, clinicians, pathologists, and imaging experts can enrich the research process by integrating diverse perspectives and expertise. By leveraging the collective knowledge of multidisciplinary teams, researchers can develop more holistic and clinically relevant solutions. Furthermore, interdisciplinary collaboration promotes the translation of research findings into practical applications, bridging the gap between scientific discovery and clinical practice. Longitudinal studies are also crucial for understanding disease progression and treatment response over time. By tracking patients’ imaging data and clinical outcomes longitudinally, researchers can elucidate the dynamic nature of *Mtb* infection and TB pathology. Lastly, investment in education and training is essential to cultivate the next generation of researchers in the field. By providing resources and opportunities for training in medical imaging, machine learning, and clinical research methodologies, we can empower researchers to tackle the complex challenges of diagnosing *Mtb* infection and TB pathology. Moreover, fostering a culture of collaboration, transparency, and open science encourages knowledge sharing and accelerates progress towards improved diagnostic methods and patient care.

## Data Availability

Data sharing is not applicable to this article as no datasets were generated or analysed during the current study.
